# Cigarette Smoking: A Modifiable Environmental Factor in the Pathogenesis of Rheumatoid Arthritis

**DOI:** 10.31662/jmaj.2025-0029

**Published:** 2025-10-03

**Authors:** Kayo Masuko

**Affiliations:** 1 Department of Internal Medicine, Karada Terrasse Ebina, Japan Medical Alliance, Ebina-shi, Kanagawa, Japan

**Keywords:** smoking, rheumatoid arthritis, autoimmunity, neo-antigens, gut microbiota

## Abstract

Cigarette smoking is increasingly recognized as a significant modifiable environmental factor in the pathogenesis of rheumatoid arthritis (RA). This review discusses the potential mechanisms through which smoking contributes to the development of RA, particularly in genetically susceptible individuals. Research has indicated that the onset of autoimmune diseases, including RA, is often preceded by a prolonged prodromal phase characterized by autoantibodies such as anti-citrullinated peptide antibodies and rheumatoid factor. Smoking has been demonstrated to increase the risk of RA, especially in individuals with specific human leucocyte antigen subtypes, and modulate the disease activity. The pathological impact of tobacco smoke may involve the induction of cellular damage and death, leading to the production of neoantigens that trigger autoimmune responses. In addition, smoking may disrupt the microbiota in the respiratory tract and intestines, which may further influence disease progression. Thus, avoiding smoking from an early age is strongly suggested in decreasing the risk of developing autoimmunity and also as a means to establish of developing preventive strategies against autoimmune diseases such as RA.

## Introduction

Autoimmune diseases such as rheumatoid arthritis (RA) and systemic lupus erythematosus (SLE) are widely recognized to develop over a “prodromal phase” lasting many years rather than appearing suddenly. Recent investigations have revealed that sera from patients with RA, which had been collected years before disease onset, were positive for the hallmark autoantibodies of RA, that is, anti-citrullinated peptide antibodies (ACPA) and rheumatoid factor (RF) ^(1), (2)^. Such findings suggest that autoimmune responses accumulate in potentially susceptible patients and that certain potential triggering event(s) in the environment exaggerate the responses to establish RA. More specifically, in individuals who possibly have a specific genetic background (e.g. the subtypes of human leucocyte antigen (HLA) including *HLA-DRB1* alleles known as “the shared epitope”) ^(3)^, environmental triggering stimuli may evoke an autoimmune cellular response. When accumulated autoimmunity crosses a certain threshold, the disease profile of RA may manifest itself. This hypothesis has been discussed and is now widely accepted under the suggested concept of “pre-RA” or “preclinical RA” ^(2), (4), (5)^.

Although this hypothesis has not been fully supported, multiple environmental factors are believed to be involved. These candidates include hormones (e.g., estradiol) and viral infections; however, studies indicate that smoking is the most important environmental factor in RA development ^(6), (7)^.

Cigarette smoking has been reported to increase the susceptibility to RA, particularly in patients with the HLA shared epitope ^(8), (9)^. A meta-analysis by Di Giuseppe et al. ^(10)^ demonstrated that, compared with nonsmokers, current smokers with RA-specific autoantibodies have an increased risk of RA (odds ratio (OR) 1.64), especially men (OR 3.91). In addition, patients with RA who smoke are more prone to seropositivity for RF or ACPA than those who do not smoke ^(11)^. Furthermore, studies showed that patients with RA who currently smoked had higher disease activity and inflammatory markers than those who smoked before or had never smoked, and that the antibody titer of ACPA was associated with cigarette smoking, especially in men ^(12), (13)^. These findings collectively support the concept that smoking is “the most common modifiable risk factor” ^(6)^ or “the best-established lifestyle risk factor” ^(7)^ for RA development.

Numerous toxic substances in tobacco smoke can chemically destroy tissues and cells in the inhalation pathways (e.g. the oral cavity, airways, and lungs), leading to local and systemic inflammation ^(14)^. However, despite the established association, the mechanism by which cigarette smoke evokes or exaggerates autoimmune diseases such as RA is unclear.

**Figure 1.**
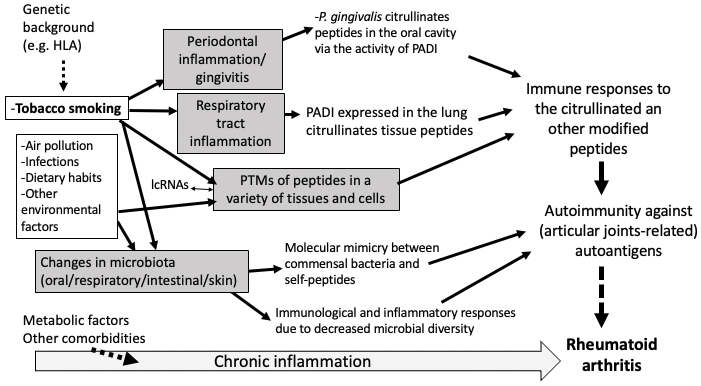
Overview of the potential roles of cigarette smoking in the induction of rheumatoid arthritis. The potential contributing pathways of cigarette smoking and other environmental factors in the pathogenesis of rheumatoid arthritis are shown. lcRNA: long non-coding RNA; PADI: peptidyl alanine deiminase; PTM: post-translational modification.

This review outlines the impact of tobacco on the development of RA from two perspectives: the induction of autoimmunity by tobacco smoke and changes in the gut microbiota caused by smoking (Figure 1).

## Cigarette Smoking and its Links to RA: Several Pathways Considered

### Cigarette smoking harms cells and tissues to produce neo-antigens

Cellular damage and death can occur when cells are exposed to toxic or harmful internal or external factors, such as infections, chemicals, or physical agents. Such cellular damage or death disrupts the cells and tissues, thus resulting in the production of neoantigens capable of evoking autoimmunity. Injured or dead cells express constituents that are not typically present in the immune system. Neoantigenic expression can initiate an autoimmune process through mechanisms such as exposure or spillage of intracellular autoantigens or the provision of autoantigenic mimics ^(15), (16), (17)^.

There are several types of cell death, including apoptosis and necrosis. Apoptosis, also known as programmed cell death, is crucial for maintaining cellular homeostasis and regulating the immune system. Abnormalities in apoptotic pathways or the clearance of apoptotic cells have been reported to contribute to the development and progression of autoimmune diseases. Apoptotic cell death can lead to the exposure or modification of self-antigens, which may trigger an autoimmune response ^(18)^. Under conditions of defective clearance of apoptotic cells, cell remnants or debris contribute to the development of autoimmunity, potentially activating the complement system and functioning as a survival signal for autoreactive B cells ^(18)^. For example, in SLE, the accumulation of post-apoptotic remnants and fragments derived from secondary necrotic cells can lead to the production of type I interferon, a hallmark of the disease ^(18)^.

In addition, recent studies have shed light on the role of neutrophil death through the formation of neutrophil extracellular traps (NETs), a process known as “NETosis”. NETs are net-like structures containing chromatin and granular enzymes that are released by a subset of activated neutrophils after interaction with invading pathogens, cytokines, and other stimuli ^(16)^. Cell death that induces the release of NETs, that is, NETosis, plays an essential role in cellular protection by clearing the invading agents. Recent studies suggest that malfunction of the NETs system may be central to the pathogenesis of a variety of autoimmune diseases. For example, NET-derived expression of damage-associated molecular patterns induces inflammatory changes in damaged tissue ^(17)^.

Cigarette smoke induces cell death in vivo. For example, exposure to tobacco smoke can trigger apoptosis in airway epithelial cells ^(19), (20)^. Additionally, smoking has been shown to increase Fas expression in B and CD4+ T lymphocytes, which may result in higher levels of apoptosis ^(21)^. Increased apoptosis leads to the exposure of intracellular citrullinated antigens, eventually breaking tolerance and inducing the production of autoantibodies ^(22)^. Furthermore, cigarette smoke has been associated with the activation of caspase-8, which contributes to pro-inflammatory responses and cell death pathways such as pyroptosis and necroptosis ^(23)^.

Cigarette smoke also induces NETosis. Nicotine, a major component of cigarette smoke, has been identified as a powerful inducer of NETosis, and nicotine exposure was reported to lead to dose-dependent NETosis in neutrophils of healthy nonsmokers ^(24)^. Certain immune complexes or inflammatory cytokines further enhance this effect, suggesting a synergistic effect of nicotine and other pro-inflammatory factors in promoting NETosis ^(23)^. Moreover, in a mouse model, serum from cigarette smoke-exposed mice showed impaired function for NETs degradation ^(25)^.

These findings suggest that smoking causes cell death and inhibits its clearance processes; therefore, smoking may be responsible for the expression and recognition of novel autoantigens produced during cell death. The findings also suggest that smoking leads to the development and exacerbation of autoimmune diseases, including RA.

### Cigarette smoking-induced post-translational protein modifications

ACPA is regarded as a representative autoantibody found in RA because its sensitivity and specificity for discriminating RA are much higher than those of RF. The target antigens for ACPA are citrullinated peptides; specifically, citrullination of arginine residues to citrulline is derived from peptidyl arginine deiminase (PADI) bioactivity. Citrullinated proteins have been reported to originate in articular joints, and local citrullination of intra-articular proteins may trigger autoantibody production in RA ^(26)^. In addition to citrullination, there are other post-translational modifications (PTM) of peptides, including carbonylation, glycation, acetylation, and glycosylation, which can alter the functions of the protein and may produce antigenic potential in the target peptides, contributing to the pathogenesis of a variety of diseases ^(1)^.

Cigarette smoking enhances the PTM of proteins. This observation is supported by various studies exploring the effect of cigarette smoke on different types of PTMs, including phosphorylation ^(27)^, carbamylation ^(28)^, and nitration ^(29)^.

Glycation is one of the processes of nonenzymatic PTM that is promoted in conditions with abnormal glucose metabolism or inflammation, such as in diabetes mellitus and RA ^(30)^. Cigarette smoking has been linked to enhanced post-translational glycation specifically through the formation of advanced glycation end products (AGEs). Research has indicated that cigarette smoke components react with plasma and extracellular matrix proteins to form covalent adducts with properties similar to those of AGEs ^(31)^. Tobacco smoke contains reactive glycation products known as “glycotoxins”, which can lead to increased AGE formation in vivo and contribute to atherosclerosis and other complications ^(32), (33)^. In vivo, smokers have higher AGE levels in many tissues than non-smokers ^(34), (35)^. Studies have shown that tobacco-derived AGEs accumulate in the biomolecules of smokers, particularly plasma low-density lipoproteins, and are thus associated with a higher risk of developing coronary and cerebrovascular diseases ^(33)^.

Glycated peptides function as autoantigens. Khan et al. ^(36)^ reported an autoimmune response to glycosylated proteins in participants of different age groups, showing that the responses were enhanced in older participants, particularly smokers. This finding may partly explain why older individuals, particularly smokers, often show positive autoantibodies and/or higher titers in their sera irrespective of the presence of clinical autoimmune diseases ^(37), (38), (39)^ and why smoking history would enhance autoimmunity via AGE formation.

Non-coding RNAs (ncRNAs) are non-protein-coding transcripts, including long non-coding RNAs (lncRNAs; >200 nucleotides), circular RNAs (cRNAs or circRNAs), and microRNAs (miRNAs; 18-25 nucleotides), expressed in various organs and tissues. Recent studies have shown that ncRNAs play important roles in gene expression and various biological response processes, even though they do not encode proteins ^(40), (41)^. The potential roles of ncRNAs in various diseases, such as autoimmune rheumatic diseases, have been attracting interest ^(40), (41)^. The possible mechanisms by which ncRNAs modulate pathogenic processes include their multifaceted influence on PTMs ^(42)^. Specifically, lnc-mediated PTMs of metabolic enzymes of the glycolytic pathway (e.g. phosphoglycerate kinase 1) and transcription factors (e.g. hypoxia-inducible factor α and nuclear factor κB) have been reported to significantly affect cellular processes and also disease progression ^(41), (42)^. Vice versa, the PTMs of proteins may modulate the expression or functions of ncRNA ^(43)^. Collectively, it can be hypothesized that smoking-induced PTMs drive the apparent expression of ncRNAs, thus leading to a vicious circle between ncRNA expression and PTMs, which accelerates the pathogenesis of various diseases, including RA. Such pathways warrant further investigation regarding autoimmunity.

### Cigarette smoking distorts the microbiome

Numerous microorganisms coexist in the human body, and with the help of these microorganisms, humans perform their daily metabolic and immune responses. Recent investigations have clarified the unexpected involvement of such microorganisms, usually those of the intestinal flora, in immune responses and the pathogenesis and pathophysiology of autoimmune diseases, including RA.

As to the terms concerning such microorganisms in vivo, ‘microbiome’ means a collection of genomes from all the microorganisms present in the environment, whereas ‘microbiota’ indicates the whole composition of microorganisms found within a specific environment.

Previous studies have shown that smoking and smoking cessation alter the human microbiome. In the following sections, these findings are briefly reviewed and summarized.

#### Changes in the oral cavity microbiome by smoking

Smoking is a major risk factor for periodontal diseases like gingivitis ^(44)^. Nicotine, chrome, and other oncogenic compounds in cigarette smoke reduce blood flow, dampen the function of white blood cells in the oral cavity, and collectively worsen oral hygiene, ultimately resulting in oral infections.

Moreover, a relationship between periodontitis and arthritis has been observed in experimental models. For example, mice with periodontitis had more severe arthritis than controls ^(45)^. In humans, it has also been reported that patients with periodontitis had a higher prevalence of RA than those without periodontitis ^(46)^.

Representative pathogenic gingivitis bacteria include *Porphyromonas* spp. such as *Porphyromonas gingivalis* and *Prevotella* spp. including *Prevotella intermedia*. Among them, *P. gingivalis* is the focus of active investigations in the pathogenesis of RA because this bacterium can uniquely produce PADI, a peptide-citrullinating enzyme ^(47)^. The enzyme-producing potential of the bacteria indicates that citrullinated peptides can be produced in the oral cavity. The modified peptides trigger an immunological reaction involving T cells on the mucosal surface, probably leading to RA development ^(45), (47)^.

Interestingly, patients with RA have been reported to have antibodies against *P. gingivalis* ^(48)^, indicating a specific immune reaction against the bacteria in vivo. Antibody production was more prominent in patients with RA and gingivitis and current smokers ^(48)^. These findings support the idea that infectious bacteria in the oral cavity trigger the autoimmune response in RA through the citrullination of peptides.

#### Smoking and microbiome in the respiratory tract

Tissues and cells in the respiratory tract, including lung epithelial, endothelial, vascular endothelial, and immune cells, are directly exposed to inhaled cigarette smoke, causing inflammation and biochemical and immune reactions. Smoking induces higher levels of pro-inflammatory cytokines and adhesion molecules, leading to chronic inflammation and deterioration of respiratory function ^(49)^.

Smoking also enhances the expression of PADI in cells such as neutrophils, monocytes, and macrophages ^(50), (51)^, suggesting that peptides in the lung can be citrullinated in situ by locally activated PADI, leading to autoimmunity-related lung damage ^(51)^.

Findings regarding air pollution may support this hypothesis, as several investigations have suggested that air pollution may increase the incidence of RA ^(52), (53)^. This hypothesis may remain controversial and require further investigation, including its implication in airway microbiota ^(54), (55)^; nevertheless, the potential role of chemical pollutants in the air, including cigarette smoke, should be regarded as a candidate triggering factor for autoimmunity in the respiratory tract.

#### Smoking modifies the gut microbiome

The human intestinal flora comprises trillions of bacteria in the gastrointestinal mucosa that control innate and adaptive immunity ^(56), (57)^. Antigenic recognition of bacterial molecules (such as flagellin or lipopolysaccharides) by toll-like receptors in host cells stimulates intracellular signaling and modulates cytokine production in the gut ^(58)^. Gut bacteria also regulate the accumulation and activity of regulatory T cells (Treg), adjusting the balance between pro-inflammatory T helper 17 cells and anti-inflammatory Tregs in the intestine ^(59), (60)^.

The diverse heterogeneity of the gut microbiota may be altered by factors such as dietary habits, intestinal or systemic inflammation, and smoking. Previous studies demonstrated that smoking reduces the heterogeneity of the gut microbiome, leading to dysbiosis ^(61), (62)^. Specifically, in the gut of smokers, the microbiome has been found to be different from that of non-smokers and shows an increase in particular taxa, including *Prevotella* ^(61), (63)^. However, smoking cessation corrected the status of the gut microbiome, similar to that of non-smokers ^(64), (65)^. Such gut dysbiosis conditions have been reported to impair gut barrier function, leading to bacterial translocation and chronic inflammation ^(66)^.

**Figure 2.**
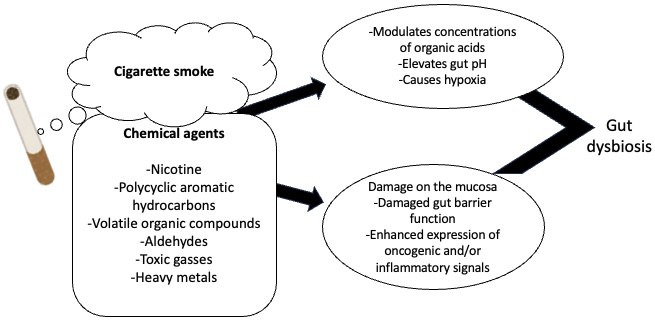
The potential mechanism by which cigarette smoking may induce gut dysbiosis.

It has been postulated that cigarette smoking alters gut microbiota diversity via several different mechanisms, both through its antimicrobial activity and modulation of the intestinal microenvironment (reviewed in ^(62), (67)^). Such pathways may include the chemical effect of toxic agents in cigarette smoke, like nicotine, the potential influence of cigarette-induced hypoxia and the resulting oxidative stress, and inflammatory changes (as summarized in Figure 2). For example, Bai et al. ^(67)^ demonstrated using a murine model that cigarette smoke induced the colonic expression of pro-inflammatory and oncogenic factors, such as tumor necrosis factor (TNF)-alpha and interleukin (IL)-17, along with the upregulation of mitogen-activated protein kinase/extracellular signal-regulated kinase(MAPK/ERK) signaling, which led to impaired gut barrier function ^(67)^.

Dysbiosis in the gut and oral cavity has been demonstrated in patients with RA and is associated with disease activity ^(68)^. A distinct bacterial taxon, *Prevotella copri*, is attracting interest because it has been predominantly found both in experimental arthritis animals and in patients with RA, even in the early phase of the disease ^(69), (70)^. Additionally, fecal transplantation from patients with RA, which included *P. copri,* induced arthritis in recipient mice ^(71)^. The precise mechanisms by which *P. copri* contributes to the pathogenesis of RA are unclear. Nevertheless, the molecular mimicry may be partially explained at least in part. There are similarities in peptide sequences between autoantigens in patients with RA and gut microbes, including *Prevotella* ^(72)^, suggesting the cross-reactivity of T cells in vivo. *P. copri*-responsive T cells have been found in the peripheral blood of patients with RA, confirming a specific immune response in vivo ^(73)^.

Collectively, these findings may support the so-called ‘mucosal origins hypothesis’, which proposes a crucial role of dysbiosis in the mucosal sites in the induction of systemic autoimmunity ^(74), (75)^. Further investigations are required to fully prove this in clinical applications, as it may be challenging to determine whether the gut microbiome is a cause or consequence of a presenting disease. The potential influence of e-cigarettes, which has been reported to ‘compromise gut triggers’, should also be carefully monitored ^(76)^. In addition, the mucosal bacterial flora and the potential influence of smoking on the skin microbiome warrant further investigation in the field of rheumatic autoimmune diseases ^(77)^.

### Increased Smoking during Quarantine due to the Pandemic: Would It Have Triggered Subsequent Autoimmunity?

Since the end of 2019, the global coronavirus disease (COVID-19) pandemic has added heavy health and economic burdens to the world. Because COVID-19 was an infectious (and potentially fatal) disease, a strict lockdown strategy was applied in several countries and regions, causing serious stress to many people under restricted physical and social movement.

We recently surveyed changes in the lifestyle and laboratory data of essentially healthy middle-aged individuals before and after the COVID-19-related quarantine in Tokyo, Japan ^(78)^. Although this was a relatively small-scale study, approximately 20% of men and 30% of women among current smokers increased their smoking habits because of the lockdown. Such an increase in smoking during the COVID-19 quarantine has also been reported in previous studies in Japan and many other countries ^(79), (80)^. This increase may be due to boredom, stress, or lack of social contact ^(81)^. Nevertheless, the potential pro-rheumatic effect of increased smoking may have further exaggerated the autoimmunity-inducing potential of COVID-19.

Not only the infection of SARS-CoV-2 itself but also the relevant vaccination has been suggested to be linked with future autoimmune rheumatic diseases ^(82), (83), (84)^. It has long been widely reported that both viral and other microbial infections can induce autoimmune diseases ^(85), (86)^, and the possible development of autoimmune rheumatic diseases due to SARS-COV-2 may represent a new example. The pathogenic mechanism has not been fully clarified, but infection with viruses, including SARS-CoV-2, or other microbes may evoke local and systemic immune reactions, causing inflammation and tissue damage, potentially inducing new self-antigens from damaged cells and tissue ^(87)^. Arthralgia is a frequent symptom of the acute phase of SARS-CoV-2 infection, and COVID-19 may be a potential cause of chronic arthritis, including RA ^(83), (88), (89)^. Nevertheless, among post-COVID-19 patients, the anti-cyclic citrullinated protein (CCP) antibody, or ACPA, a hallmark autoantibody in RA, did not show any significant differences between the patients with and without arthritis ^(89)^; therefore, cytokine dysregulation, rather than antibody-mediated responses, may play a role in the occurrence of RA following COVID-19 ^(82), (89)^. Of note, the suggested similarity of macrophages in the lungs (alveolar macrophages) and synovial tissue macrophages may introduce a common mechanistic pathway of inflammation between COVID-19 and RA ^(88)^.

**Figure 3.**
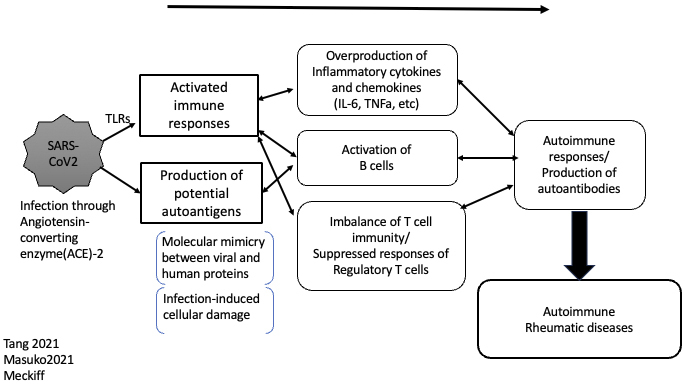
Suggested mechanisms of the induction of autoimmune rheumatic diseases by SARS-CoV-2 infection. SARS-CoV-2: severe acute respiratory symdrome-coronavirus-2.

Thus, SARS-CoV-2 infection can cause or trigger rheumatic diseases (Figure 3); therefore, in addition to developing safer vaccines and vaccination recommendations aimed at appropriate targets, social or public health measures to prevent infection will be important not only regarding the infection itself but also in the prevention of possible future autoimmune diseases.

## Concluding Remarks: Where There is Smoke, There May Be RA

Some environmental pathogenic factors that have been suggested to cause RA are avoidable. The first factor is cigarette smoking, which triggers and enhances autoimmune and inflammatory responses in the respiratory and intestinal tracts (Figure 1). Moreover, accumulating data suggest that passive smoking during childhood has a direct influence on the incidence of RA ^(90)^.

**Figure 4.**
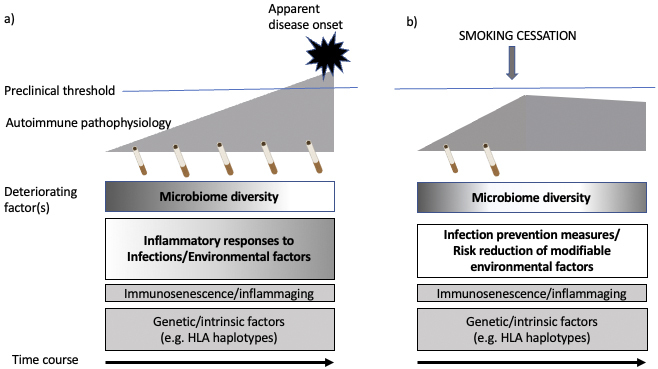
The potential roles of cigarette smoking in the induction of autoimmunity. a) Implication of cigarette smoking in the induction of apparent autoimmunity. b) Cessation of cigarette smoking may suppress the progression of autoimmune responses partly through the recovery of diversity of the microbiome.

Considering that autoimmune responses may occur years before the first manifestation of rheumatic symptoms and that there is no way for future patients to know the latent signal at that time, not smoking cigarettes in the first place or quitting smoking as early as possible would be important for preventing a further cascade of autoimmune responses and inflammatory changes (Figure 4).

Future investigations to assess how smoking cessation could prevent the progression from pre-RA to overt RA are urgently needed.

## Article Information

### Author Contribution

The author solely conceived, designed, and wrote this review article. All aspects of the work, including literature review, analysis, and manuscript preparation, were carried out independently by the author.

### Conflicts of Interest

None

### IRB Approval Code and Name of the Institution

Not applicable.
